# Revisiting old lessons from classic literature on persistent global pollutants: *This article belongs to* Ambio*’s 50th Anniversary Collection. Theme: Environmental contaminants*

**DOI:** 10.1007/s13280-020-01413-w

**Published:** 2021-01-19

**Authors:** Jonathan W. Martin

**Affiliations:** Science for Life Laboratory, Department of Environmental Science, Stockholm University, 106 91, Stockholm, Sweden

**Keywords:** Chemical regulation, Environmental contaminants, Mercury, Persistent, Polychlorinated

## Abstract

Looking back 50 years at classic literature was a reminder of inspiring discoveries and clever theories that were formative to the field of environmental chemistry, but also of the irreparable costs that persistent global pollutants have had on ecosystems and human society. In my view, these three papers have greatly impacted contemporary science and influenced development of policies that have limited the spread of hazardous contaminants. At the same time, a sobering reality is that reversing decades of past pollution has proven impossible in our lifetime, and global trends are dire for both legacy and emerging contaminants. Lessons in these papers are clear to most environmental scientists, but I argue have not resulted in adequate investment in infrastructure or manpower to enable systematic unbiased searching for pollutants as proposed by Sören Jensen in 1972. Acknowledging that the costs of new global contaminants will be too high, we must incentivize safer chemicals and their sustainable use, increase international exchange of lists of chemicals in commerce, and coordinate international efforts in nontarget screening to identify new contaminants before they circulate the world.

## Inspiring science and stories that remain highly relevant

It is with enormous humility that I offer my perspective on these three classic articles, all of which are authored by some of my personal heroes in environmental science. Each paper contains important scientific principles, but also surprising stories of irreparable contamination that inspired me as a student and compelled me towards a research career. After so many years, these principles and stories remain relevant core material that I still teach today to inspire and educate the next generation of environmental chemists and toxicologists. In fact, I was not even born at the time Sören Jensen contributed his seminal *PCB Story* from Stockholm University (Jensen 1972), and the chemical transport mechanisms of *Global Fractionation and Cold Condensation* (Wania and Mackay 1993) were already solidified as textbook material by the time of my undergraduate education in Canada, one of 8 circumpolar countries highly impacted by persistent pollutants in Arctic wildlife and northern communities. I do not take it for granted that subsequent opportunities for me to perform graduate research on persistent organic contaminants, or later to work as a professor in Canada and Sweden, may never have arisen if not for these pioneering works.

It was also during my undergraduate education that I first learned about the high exposure of Canadian communities to methyl mercury (MeHg), and of its disturbing developmental toxicity as described by Mergler et al. (2007). In their highly cited paper, they remind us that MeHg exposure is now a worldwide concern. Sadly, this reminder came more than 50 years after the important lessons of Minimata Bay, Japan, when irreversible neurodevelopmental effects were first noted among children of mothers exposed to high MeHg in seafood, contaminated by local industrial effluent. These neurodevelopmental symptoms are called Minimata disease, but diagnoses are no longer limited to Japan. A lesser known but similarly devastating example occurred throughout the 1960s and 1970s in Ontario, Canada, where paper mill effluents containing mercury were continually released to the English-Wabigoon river system upstream of the Canadian Grassy Narrows First Nation. Subsequent exposure of the community to MeHg is now considered one of Canada’s worst environmental disasters. Many people in the small community have been formally diagnosed with Minimata disease, and others struggle with persistent subclinical symptoms (Philibert et al. 2020). Despite taking place in one of the wealthiest democracies in the world, the mercury pollution has not been cleaned up and there is ongoing suffering.

As noted by Wania and Mackay (1993), those people with highest exposures to persistent organic pollutants (POPs) in the Arctic are not the same people who enjoyed many of the economic or lifestyle benefits associated with their historic use at southern latitudes. Such social injustice is a common pattern in cases of environmental pollution, and this exacerbates social inequity while damaging culture and tradition. As illustrated above for MeHg contamination, the people of Grassy Narrows had little opportunity to reduce their exposure without giving up their traditions of hunting and fishing on native lands. Switching from traditional foods to commercial foods often results in poorer nutrition and creates a new financial burden, particularly for remote communities. Despite great leaps in scientific understanding, stories like these keep me aware of the underlying human and environmental costs these classic contaminants continue to impart on the world.

## Impact on policy, but persistent limitations

Without a doubt, one of the greatest impacts of these classic papers is their influence on development of chemical regulatory policy at domestic and international levels. Sören Jensen reported his discovery of PCBs in 1966, and by 1972 Sweden had already enacted a law that prohibited the use, manufacture, or import of PCBs. Similar regulatory measures occurred soon thereafter in many developed countries. Nevertheless, persistent and mobile pollutants require global action, and such policies understandably took many more years to be negotiated and ratified by hundreds of countries. The international Stockholm Convention on POPs was not adopted until 2001, and the Minimata Convention on mercury was only recently adopted, in 2013. These international agreements are incredible milestone achievements for society, and testament that scientific knowledge can lead to informed policy decisions at the highest level of governance. While these are clearly scientific and political success stories, it is important to keep in mind that these policies are primarily designed to prevent environmental contamination from getting worse. Whether or not these policies have, or will, lead to reduced global contamination remains dubious. This is an important reminder that global pollutants should be prevented proactively, as they cannot necessarily be remediated, even on the time scale of generations. In this context, there is awareness that persistent global pollutants are potential *planetary boundary threats* which threaten the safe operating space for humanity (Persson et al. 2013).

## Dire global trends

For the foreseeable future of the Arctic, a legacy of contamination by POPs and mercury is here to stay. The most recent Arctic Monitoring and Assessment Report (AMAP 2018) notes that despite today’s policies, concentrations of these chemicals in many Arctic top predators remain elevated and may no longer be declining. There is high concern for polar bears, whales, seals, and various birds with regard to risks of immune, reproductive, or carcinogenic effects. In my research group’s hunt for new fluorinated contaminants in the Arctic, we accidentally stumbled on new classes of major PCB metabolites in polar bears using nontarget mass spectrometry (Liu et al. 2018). Thus, more than 50 years later ‘The PCB Story’ continues to unfold by incremental serendipitous discovery, and there are still unanswered questions about how PCBs affect health of key Arctic species. As the number of contaminants detected in tissues of Arctic organisms has increased into the hundreds, the mixture-effect becomes a more relevant toxicological question which is only recently starting to be investigated (Desforges et al. 2017).

Global levels and trends in mercury contamination are also cause for high concern. Although MeHg concentrations in freshwater fish slowly declined beginning in 1972, trends appear to be upward today because of increasing global mercury emissions starting in the early 1990s (Grieb et al. 2019). While MeHg concentrations in commercially relevant marine fish from the North Atlantic are declining, concentrations are increasing in the North Pacific because of increased mercury loadings to that region (Grieb et al. 2019). Considering the human health risk, a troubling fact is the overall increase in dietary intake of MeHg over the last 50 years (1960–2011). In one key study, 66 of 175 countries exceeded the recommended tolerable intake of MeHg, and many of the worst impacted countries are small island developing states, and/or least developed countries (Lavoie et al. 2018), another example of the social injustice posed by pollution.

At mid-latitudes, there is relatively good news for POPs in response to enacted policies. In the Great Lakes region of North America, atmospheric PCB concentrations decreased 3–10%/year between 1990 and 2003, lake water concentrations declined by 8% in Lakes Superior(1970–1995) and Michigan (1980–1991), and PCBs in herring gull eggs and predatory fish showed significant decreases, as summarized by Chang and colleagues (Chang et al. 2012). Studies in North America and Europe show PCB concentrations have also declined slowly in people, for example in Germany infant intake of PCBs and chlorinated POPs in breast milk declined considerably (e.g. 5–10*x*) from 1985 to 2003. However, at the same time the concentration of polybrominated diphenyl ethers (PBDEs) nearly doubled in human milk between 1992 and 2002 (Furst 2006).

## Impact on contemporary science

In addition to their impact on policy, the careful methods of contaminant discovery for PCBs (Jensen 1972), the deep thinking about environmental transport of chlorinated POPs (Wania and Mackay 1993), and the meticulous exposure and effect characterizations compiled for MeHg (Mergler et al. 2007) are now benchmarks and models that have had a lasting influence on the way modern environmental chemistry and health studies are hypothesized, conducted, or reported. One can easily find parallels between the PCB Story, ultimately enabled by new availability of sensitive GC–MS technology at the time, and the more contemporary tale of perfluoroalkyl acid discovery in humans (Hansen et al. 2001) and wildlife (Kannan et al. 2001), ultimately enabled by LC-electrospray tandem mass spectrometers. Jensen also described how high blank levels and unavailable pure standards hampered the pace of his discoveries, but he overcame these in ways similar to what was described much later for perfluoroalkyl substance discoveries (Martin et al. 2004). When polybrominated diphenyl ethers were observed to be increasing in Arctic wildlife, the cold condensation theories of Wania and Mackay were immediately applicable to explain this (Ikonomou et al. 2002). For perfluoroalkyl acids, which have no measurable vapour pressure, realization that other important transport mechanisms must be operational at the global scale led to new hypotheses, such as the search for volatile precursors (Martin et al. 2002; Ellis et al. 2004). Later it was shown that perfluorooctanoic acid was a good ‘swimmer’ (Wania 2006), capable of reaching the Arctic in slow marine currents (Armitage et al. 2009).

Methyl mercury is now considered a classic neurodevelopmental toxicant, but emerging contaminants are now being considered with the same attention to global exposure patterns, toxicological and epidemiological evidence of developmental effects, such as for PBDEs, perfluoroalkyl acids, phthalates, and bisphenols. For all of these, research attention has turned to understanding the mechanisms which might explain the persistent neurodevelopmental deficits of offspring after in utero exposure, with specific attention to epigenetic mechanisms (Tran and Miyake 2017). Contaminant exposures that we experienced in early life may not only affect our own health, but may also predispose our children, or our children’s children to higher risks of diseases in their respective lifetimes. These additional costs of past environmental pollution have yet to be fully accounted for. Mergler et al. also discussed the complications of human co-exposure to MeHg and PCBs through dietary fish in the context that these may confound human studies or interact together toxicologically (Mergler et al. 2007). Fish is now also recognized as the main source of exposure to many perfluorinated compounds, such as perfluorooctane sulfonate (PFOS). It will never be known to what extent PFOS may have confounded past health studies of MeHg or PCBs, but one study has shown that PFOS and MeHg can interact to affect brain biochemistry and behaviour of rodents exposed in utero (Reardon et al. 2019).

## Have we learned our lessons?

Notwithstanding the great scientific impact of these papers and their eventual influence on domestic and international policy, other opportunities remain to be realized. These articles contained new discoveries, new concepts, and clear summaries of multidisciplinary knowledge at the time of their writing, and in each case there are important lessons that should have altered society. While we have understood the scientific principles, and built on these over the years, in my opinion the morals of these stories have failed to have the impact on society that was deserved. I am concerned that the great human and environmental costs of environmental contamination have not been fully accounted for or understood, and the type of transformative change called for 50 years ago has yet to be fully heeded. In conclusion to his 1972 article describing the serendipitous discovery of PCBs, Sören Jensen tells us the moral of his story in concise and prophetic statements which deserve repeating:

The accumulation of PCB in nature was discovered accidentally, as was mercury contamination… Similar discoveries of the accumulation of other chemicals are quite likely to occur at any time… It is necessary that responsible authorities invest in manpower and equipment to facilitate an unbiased search for pollutants at an early stage by systematic analysis. These are the measures that should be taken if the damaging and perhaps irremedial effects of a substance are to be discovered before and not after it has entered the environment. (Jensen 1972)

The lesson was clear and the suggestion was wise, but so far we have failed as a society to adequately invest and achieve the vision of Sören Jensen. New contaminant discoveries have occurred slowly, either accidentally or due to hypothesis driven ideas of individuals, rather than through any systematic, unbiased, or coordinated approach. While there are international frameworks and networks for systematic monitoring of existing contaminants, there are still no ongoing initiatives to screen in a coordinated way for new persistent contaminants emerging from today’s source regions.

## So, what now?

We cannot stop the important scientific work we are already doing to understand known contaminants, but increased efforts aimed at preventing future persistent contaminants from circulating globally should be a priority for our field today. This will require major coordination across countries and new injections of funding, which can easily be justified if only a few classes of new chemicals are detected early and prevented from becoming tomorrow’s new POPs. If the political will can be found, the good news is that powerful tools and approaches in nontarget mass spectrometry are well suited to the task at hand. High-frequency full-scanning ultrahigh resolution mass spectrometers with GC or LC inlets are capable of unbiased discovery of contaminants in complex samples. To aid in the task, over the last 15 years comprehensive lists of chemicals in commerce in the US, Canada, and Europe (~ 350 000 chemicals) have been compiled and screened with in silico tools to prioritize environmental monitoring. In a promising development, this year these efforts were extended to include China, and many unique persistent and bioaccumulative chemicals have now been highlighted (Zhang et al. 2020). Such efforts must continue, with cooperation still needed from major countries of South Asia and of the Southern Hemisphere. Other public online tools are also available which can be harnessed to coordinate international cooperation, including vast public chemical databases with descriptive metadata (e.g. Comptox Chemicals Dashboard), free high-resolution mass-spectral libraries (e.g. massbank.eu), and several open-science data-processing software.

Other aspects of society which must improve are more sustainable chemical use, and to incentivize the development of less hazardous chemicals by industry. By 2008, scientists already had the ability to screen long lists of existing chemicals in commerce for their potential to be POPs based on measured or estimated physical properties (Brown and Wania 2008), but now the same knowledge should be turned upside-down and be used to innovate a new generation of chemicals without hazardous properties of POPs. In a rational and sustainable future, old problematic chemicals should be replaced with new chemicals that are fit for purpose and ‘benign by design’ (Anastas 1994). In an essay in *Science* (Collins 2001), such development was long-ago deemed a priority, yet the authors acknowledged that educational curriculums in chemistry were still deficient. Organic chemistry students will learn that chlorination of phenol proceeds by electrophilic aromatic substitution, but will they also learn that chlorinated phenols are persistent or may act as endocrine disruptors? It is not obvious to me that these challenges have yet been met.

I am nevertheless optimistic that these systemic changes can be made, even though they are already decades overdue. By looking to the past at these classic papers, and thereby acknowledging the harm and great financial and social costs of today’s global pollutants, we can look forward with vigour to prevent future generations of POPs. Akin to slowing progression of the current Covid-19 pandemic, or bending the curve of CO_2_ emissions to limit global warming, the road map to prevent new global contamination is clear but will require global cooperation and increased public education on the effects and threats of persistent global pollutants.

## References

[CR1] AMAP. 2018. Biological effects of contaminants on Arctic wildlife & fish. AMAP Assessment Report 2018. Summary for Policy Makers. https://www.amap.no/documents/doc/biological-effects-of-contaminants-on-arctic-wildlife-and-fish.-summary-for-policy-makers/1763.

[CR2] Anastas PT (1994). Benign by design chemistry. Benign by Design.

[CR3] Armitage JM, MacLeod M, Cousins IT (2009). Modeling the global fate and transport of perfluorooctanoic acid (PFOA) and perfluorooctanoate (PFO) emitted from direct sources using a multispecies mass balance model. Environmental Science and Technology.

[CR4] Brown TN, Wania F (2008). Screening chemicals for the potential to be persistent organic pollutants: A case study of Arctic contaminants. Environmental Science and Technology.

[CR5] Chang F, Pagano JJ, Crimmins BS, Milligan MS, Xia X, Hopke PK, Holsen TM (2012). Temporal trends of polychlorinated biphenyls and organochlorine pesticides in Great Lakes fish, 1999–2009. Science of the Total Environment.

[CR6] Collins T (2001). Essays on science and society. Toward sustainable chemistry. Science.

[CR7] Desforges JP, Levin M, Jasperse L, De Guise S, Eulaers I, Letcher RJ, Acquarone M, Nordoy E (2017). Effects of polar bear and killer whale derived contaminant cocktails on marine mammal immunity. Environmental Science and Technology.

[CR8] Ellis DA, Martin JW, De Silva AO, Mabury SA, Hurley MD, Sulbaek Andersen MP, Wallington TJ (2004). Degradation of fluorotelomer alcohols: A likely atmospheric source of perfluorinated carboxylic acids. Environmental Science and Technology.

[CR9] Furst P (2006). Dioxins, polychlorinated biphenyls and other organohalogen compounds in human milk - Levels, correlations, trends and exposure through breastfeeding. Molecular Nutrition & Food Research.

[CR10] Grieb TM, Fisher NS, Karimi R, Levin L (2019). An assessment of temporal trends in mercury concentrations in fish. Ecotoxicology.

[CR11] Hansen KJ, Clemen LA, Ellefson ME, Johnson HO (2001). Compound-specific, quantitative characterization of organic fluorochemicals in biological matrices. Environmental Science and Technology.

[CR12] Ikonomou MG, Rayne S, Addison RF (2002). Exponential increases of the brominated flame retardants, polybrominated diphenyl ethers, in the Canadian Arctic from 1981 to 2000. Environmental Science and Technology.

[CR13] Jensen S (1972). The PCB Story. Ambio.

[CR14] Kannan K, Koistinen J, Beckmen K, Evans T, Gorzelany JF, Hansen KJ, Jones PD, Helle E (2001). Accumulation of perfluorooctane sulfonate in marine mammals. Environmental Science and Technology.

[CR15] Lavoie RA, Bouffard A, Maranger R, Amyot M (2018). Mercury transport and human exposure from global marine fisheries. Scientific Reports.

[CR16] Liu Y, Richardson ES, Derocher AE, Lunn NJ, Lehmler HJ, Li X, Zhang Y, Cui JY (2018). Hundreds of unrecognized halogenated contaminants discovered in polar bear serum. Angewandte Chemie (International ed. in English).

[CR17] Martin JW, Kannan K, Berger U, de Voogt P, Field J, Franklin J, Giesy JP, Harner T (2004). Analytical challenges hamper perfluoroalkyl research. Environmental Science and Technology.

[CR18] Martin JW, Muir DC, Moody CA, Ellis DA, Kwan WC, Solomon KR, Mabury SA (2002). Collection of airborne fluorinated organics and analysis by gas chromatography/chemical ionization mass spectrometry. Analytical Chemistry.

[CR19] Mergler D, Anderson HA, Chan LH, Mahaffey KR, Murray M, Sakamoto M, Stern AH, R. Panel on Health and M. Toxicological Effects of (2007). Methylmercury exposure and health effects in humans: A worldwide concern. Ambio.

[CR20] Persson LM, Breitholtz M, Cousins IT, de Wit CA, MacLeod M, McLachlan MS (2013). Confronting unknown planetary boundary threats from chemical pollution. Environmental Science and Technology.

[CR21] Philibert A, Fillion M, Mergler D (2020). Mercury exposure and premature mortality in the Grassy Narrows First Nation community: A retrospective longitudinal study. Lancet Planet Health.

[CR22] Reardon AJF, Karathra J, Ribbenstedt A, Benskin JP, MacDonald AM, Kinniburgh DW, Hamilton TJ, Fouad K (2019). Neurodevelopmental and metabolomic responses from prenatal coexposure to perfluorooctanesulfonate (PFOS) and methylmercury (MeHg) in Sprague-Dawley rats. Chemical Research in Toxicology.

[CR23] Tran NQV, Miyake K (2017). Neurodevelopmental disorders and environmental toxicants: Epigenetics as an underlying mechanism. International Journal of Genomics.

[CR24] Wania F (2006). Potential of degradable organic chemicals for absolute and relative enrichment in the Arctic. Environmental Science and Technology.

[CR25] Wania F, Mackay D (1993). Global fractionation and cold condensation of low volatility organochlorine compounds in polar-regions. Ambio.

[CR26] Zhang X, Sun X, Jiang R, Zeng EY, Sunderland EM, Muir DCG (2020). Screening new persistent and bioaccumulative organics in China’s inventory of industrial chemicals. Environmental Science and Technology.

